# Trends in Serum Lipid Levels of a 10- and 13-Year-Old Population in Fukuroi City, Japan (2007–2017)

**DOI:** 10.2188/jea.JE20180164

**Published:** 2020-01-05

**Authors:** Katsuyasu Kouda, Masayuki Iki, Yuki Fujita, Harunobu Nakamura, Kumiko Ohara, Takahiro Tachiki, Toshimasa Nishiyama

**Affiliations:** 1 Department of Hygiene and Public Health, Kansai Medical University, Osaka, Japan; 2 Department of Public Health, Kindai University Faculty of Medicine, Osaka, Japan; 3 Department of Health Promotion and Education, Graduate School of Human Development and Environment, Kobe University, Kobe, Japan

**Keywords:** child, cholesterol, epidemiology, lipoproteins

## Abstract

**Background:**

Current trends in serum lipid levels among children are likely to be important predictors of future cardiovascular disease prevalence. However, no studies have examined trends in low-density lipoprotein cholesterol (LDL-C) levels in Japanese children.

**Methods:**

We investigated trends in LDL-C levels from 2008 through 2017 and HDL-C levels from 2007 through 2017 in a population of 10- and 13-year-old children in Fukuroi City, Japan. We analyzed 17,838 children, accounting for 93.8% of all fifth and eighth graders in the entire city. Adverse lipid levels were defined as follows: 130 mg/dL or higher for LDL-C, and lower than 40 mg/dL for HDL-C. The Jonckheere-Terpstra and Cochran-Armitage tests were used to evaluate secular trends in mean serum lipid levels and prevalence of dyslipidemia, respectively.

**Results:**

There were no significant trends in BMI during the study period. In children aged 10 years, serum levels of LDL-C and HDL-C showed significant positive associations with calendar year during the study period for both sexes. A significant increase in HDL-C levels was observed in girls aged 13 years. On the other hand, no significant trends were observed in the prevalence of high LDL-C or low HDL-C regardless of sex or age, while the prevalence of high non-HDL-C showed a significant increase in boys.

**Conclusions:**

In the Fukuroi population, serum levels of LDL-C and HDL-C slightly increased in both boys and girls aged 10 years, and HDL-C levels slightly increased in girls aged 13 years, during the past decade.

## INTRODUCTION

Elevated low-density lipoprotein cholesterol (LDL-C) has been shown to be a major cause of coronary heart disease in epidemiological studies, as well as in animal and laboratory experiments.^1^ Carotid arterial wall atherosclerosis rapidly progresses during childhood in individuals with familial hypercholesterolemia, which is characterized by elevated LDL-C.^2^ It is widely acknowledged that children initially identified as having elevated levels of serum cholesterol tend to have elevated follow-up levels (in studies tracking childhood to adulthood).^3^^–^^8^ Adverse LDL-C levels in childhood persist over time, progressing to dyslipidemia in adulthood.^8^ A population-based, prospective cohort study conducted in Finland showed an association between adult carotid artery intima-media thickness (IMT) and childhood LDL-C levels.^9^ A cohort study of a semirural black and white community in Bogalusa, Louisiana, United States also demonstrated that childhood LDL-C levels predict carotid IMT in young adults.^10^ Moreover, intimal atherosclerotic lesions reportedly appeared in the aortas and coronary arteries of adolescent individuals who died of external causes and underwent autopsy.^11^ In Japan, a nationwide autopsy study of atherosclerosis in infants, children, and young adults found that atherosclerotic lesions undergo substantial developments in extent and prevalence between 10 and 39 years of age.^12^^,^^13^ Thus, the influence of risk factors in early life may contribute to the development of future cardiovascular disease.^9^ The Expert Panel on Integrated Guidelines for Cardiovascular Health and Risk Reduction in Children and Adolescents strongly recommends a universal lipid screening in 9- to 11-year-old children to prevent future cardiovascular disease.^14^ Current trends in childhood cholesterol levels might predict subsequent cardiovascular disease trends in adults.

In the United States, beneficial trends in serum lipid concentrations were reportedly observed among youths between 1988–1994 and 2007–2010, while adverse lipid profiles persisted.^15^ Adverse trends in serum lipid concentrations were also reported among Chinese children during a period from 2004 to 2014.^16^ In Japan, nationwide surveys on serum total cholesterol (T-C) were conducted in 1960, 1970, 1980, and 1990, revealing a yearly increase in T-C concentration among 10- to 19-year-old males and females.^17^ However, since T-C concentration reflects both levels of LDL-C and high-density lipoprotein cholesterol (HDL-C), which is known as good cholesterol,^18^ studies that directly evaluate secular trends in LDL-C and HDL-C levels among children are warranted. We recently reported that there were no significant trends in T-C, non-HDL-C, and HDL-C levels in a 10-year-old population in Iwata City, Shizuoka, Japan from 1993 to 2008.^19^ Since that study was conducted, there have been no reports on serum lipid trends in Japanese children. Moreover, no previous study has examined secular trends in LDL-C among Japanese schoolchildren. Accordingly, the present study aimed to provide the most up-to-date data regarding serum lipid levels in children and investigated secular trends in LDL-C levels from 2008 through 2017 and HDL-C levels from 2007 through 2017 in a 10- and 13-year-old population in Fukuroi City, Shizuoka, Japan.

## METHODS

### Study population

Fukuroi City, in Shizuoka Prefecture, Japan, is located at 135 degrees east longitude and 34 degrees north latitude, roughly 240 km away from Tokyo and 320 km from Osaka, and covers an area of 108 km^2^. Fukuroi City is adjacent to Iwata City, where the trends in serum T-C from 1993 to 2001^20^ and HDL-C from 1993 to 2008^19^ were previously reported. Fukuroi City had a population of 87,174 as of April 2016. Land use consisted of 17.2% residential, 36.2% agricultural, and 19.5% mountainous woodlands in 2011. Approximately 4% of the labor force is engaged in primary industries, while 40% and 53% are engaged in secondary and tertiary industries, respectively. Both commerce and industry in the city have developed proportionately with the development of transport lines, such as National Route 1, Tomei expressway, Tokaido Shinkansen Line, and Tokaido Main Line.

Fukuroi City has 12 municipal elementary schools, 4 municipal junior high schools, and no private schools. Almost all children living in the city are enrolled in municipal schools. There were 19,008 fifth and eighth grade children who attended municipal schools from 2007 through 2017. The Fukuroi Board of Education conducts health screening including anthropometry measurements and blood tests for all fifth and eighth graders during April through June every year. The source population of the present study comprised 19,008 children; of these, 17,838 who participated in health examinations from 2007 through 2017 were included in the analysis. The present study protocol was approved by the Ethics Committee of the Kindai University Faculty of Medicine. This study was conducted in accordance with the ethical principles of the Declaration of Helsinki.

### Examinations

Anthropometric variables were measured in each school during April through June each year by *Yogo* teachers who have a Japan national health education license. Measurements were conducted in accordance with the Japanese School Health and Safety Act to ensure uniform quality control. Height was measured to an accuracy of 0.1 cm, and body weight to 0.1 kg.^21^ Body mass index (BMI; kg/m^2^) was calculated as body weight (kg) divided by height squared (m^2^). To determine overweight subjects, cut-offs of BMI based on an adult BMI of 25 kg/m^2^ were calculated using the standardized centile curves of BMI for Japanese children.^22^ Cut-offs for boys and girls aged 10 years were >20.04 kg/m^2^ and >20.97 kg/m^2^, respectively, and those for boys and girls aged 13 years were >23.26 kg/m^2^ and >23.89 kg/m^2^, respectively. Similarly, cut-offs of BMI based on an adult BMI of 18.5 kg/m^2^ were calculated to determine underweight subjects. Cut-offs for boys and girls aged 10 years were <14.57 kg/m^2^ and <14.39 kg/m^2^, respectively, and those for underweight boys and girls aged 13 years were <16.52 kg/m^2^ and <16.17 kg/m^2^, respectively.

Non-fasting blood samples were collected by nurses and medical technologists who were staff members of the *Shizuokaken Yoboigakukyokai* (the Shizuoka Prefecture Preventive Medicine Association, Shizuoka, Japan) in each school during April through June each year. LDL-C and HDL-C levels were measured using the direct method with commercial assays (Cholestest LDL and Cholestest N HDL, Sekisui Medical Co. Ltd., Tokyo, Japan, from 2007 through 2010; Determiner L LDL-C and MetaboLead HDL-C, Kyowa Medex Co. Ltd., Tokyo, Japan, from 2011 to 2017). Intra- and inter-laboratory coefficients of variation were less than 4% throughout the study period (2007–2017). Cut-offs for high LDL-C and low HDL-C were defined according to the 2011 Expert Panel on Integrated Guidelines for Cardiovascular Health and Risk Reduction in Children and Adolescents as follows: high LDL-C, ≥130 mg/dL; high non-HDL-C, ≥145 mg/dL; and low HDL-C, <40 mg/dL.^14^

### Statistical analysis

Statistical analysis was performed with SAS software for Windows ver. 9.4 (SAS Institute Inc., Cary, NC, USA). The Jonckheere-Terpstra test was used to evaluate secular trends in anthropometric variables and levels of serum lipids during the study period. The explanatory variable was the calendar year of health examination. Dependent variables were individual measurements of height, body weight, BMI, LDL-C, non-HDL-C, HDL-C, T-C, and the ratio of LDL-C to HDL-C. In addition, the Cochran-Armitage test was used to evaluate secular trends in prevalence of overweight, underweight, high LDL-C, high non-HDL-C, and low HDL-C. Since differences in serum lipid levels are known to be associated with age and sex,^23^ sex- and age-specific evaluations were performed in each regression model. *P* < 0.05 was considered statistically significant.

## RESULTS

Table 1 shows anthropometric variables over the period from 2007 through 2017. Although height and weight in girls aged 10 years showed significant increases, there were no significant trends in BMI in both boys and girls aged 10 and 13 years during the study period. No significant trends were observed in the prevalence of overweight in both boys and girls aged 10 and 13 years during the study period, while a significant decrease was observed in girls aged 10 years who were judged to be underweight (Table 2).

**Table 1.** Secular trends in anthropometric measurements

	Number of children	Height			Weight			BMI			Number of children	Height			Weight			BMI		
					
		Percentiles										Percentiles								
	
		5th	50th	95th	5th	50th	95th	5th	50th	95th		5th	50th	95th	5th	50th	95th	5th	50th	95th
	Boys at age 10										Girls at age 10									
	
2007	387	128	138	149	25	32	45	14	17	23	400	127	138	150	24	32	44	14	16	21
2008	430	129	138	149	25	32	47	14	17	23	391	129	138	150	25	32	45	14	17	22
2009	452	129	138	149	25	32	47	14	17	23	380	129	139	150	25	32	46	14	17	22
2010	419	129	138	148	26	32	45	14	17	23	432	128	139	150	24	32	45	14	17	21
2011	461	129	138	146	26	31	47	14	17	23	395	128	139	150	25	32	46	14	17	22
2012	423	128	138	148	26	32	47	15	17	23	420	128	139	151	25	32	46	14	17	21
2013	434	127	138	148	25	32	50	14	17	23	410	129	139	150	25	32	46	14	17	22
2014	435	128	139	149	25	32	46	14	17	23	434	128	139	151	25	32	46	14	17	21
2015	442	130	138	148	26	32	48	15	17	23	420	128	139	150	25	32	44	14	17	21
2016	430	128	138	148	25	32	47	14	17	23	395	128	139	152	25	32	49	14	17	22
2017	502	128	138	149	25	33	49	14	17	24	412	129	140	152	25	33	47	14	17	22
*P* for trend		0.324			0.120			0.143				0.005			0.029			0.216		

	Boys at age 13										Girls at age 13									
	
2007	395	145	160	171	35	47	70	16	18	26	373	145	155	163	37	46	60	16	19	24
2008	382	146	160	171	35	47	65	16	18	24	346	146	155	163	37	46	60	16	19	25
2009	389	145	159	170	35	47	66	16	19	25	360	146	154	162	37	46	58	16	19	24
2010	360	145	160	171	35	48	65	16	18	23	387	145	154	163	35	46	58	16	19	24
2011	406	146	159	171	35	47	63	16	18	23	372	145	153	163	37	46	59	16	19	24
2012	434	146	159	170	35	48	65	16	18	24	356	145	154	162	36	45	60	16	19	25
2013	388	147	159	170	36	47	62	16	19	24	404	146	154	162	36	45	58	16	19	23
2014	424	147	158	169	35	46	63	16	18	23	383	145	154	162	36	46	59	16	19	24
2015	378	145	159	171	35	47	63	16	18	23	395	145	154	163	36	46	59	16	19	24
2016	403	145	160	171	34	47	65	16	18	25	374	146	155	162	36	46	59	16	19	24
2017	409	146	160	172	35	47	66	16	18	25	416	145	154	163	36	46	60	16	19	25
*P* for trend		0.335			0.568			0.097				0.585			0.296			0.206		

BMI, body mass index.

The Jonckheere-Terpstra test was used to evaluate secular trends, with calendar year as the explanatory variable and individual measurements as dependent variables.

**Table 2.** Secular trends in overweight and underweight children (numbers and proportions)

	Total	Overweight		Underweight		Total	Overweight		Underweight	
	Boys at age 10					Girls at age 10				
	
2007	387	52	13.4%	25	6.5%	400	27	6.8%	32	8.0%
2008	430	61	14.2%	24	5.6%	391	29	7.4%	26	6.6%
2009	452	56	12.4%	32	7.1%	380	29	7.6%	35	9.2%
2010	419	50	11.9%	29	6.9%	432	20	4.6%	37	8.6%
2011	461	52	11.3%	45	9.8%	395	30	7.6%	20	5.1%
2012	423	50	11.8%	22	5.2%	420	26	6.2%	29	6.9%
2013	434	67	15.4%	31	7.1%	410	29	7.1%	33	8.0%
2014	435	54	12.4%	36	8.3%	434	23	5.3%	19	4.4%
2015	442	59	13.3%	26	5.9%	420	25	6.0%	23	5.5%
2016	430	60	14.0%	30	7.0%	395	38	9.6%	24	6.1%
2017	502	81	16.1%	36	7.2%	412	29	7.0%	27	6.6%

*P* for trend		0.182		0.686			0.645		0.044	

	Boys at age 13					Girls at age 13				
	
2007	395	36	9.1%	61	15.4%	373	21	5.6%	17	4.6%
2008	382	26	6.8%	55	14.4%	346	24	6.9%	24	6.9%
2009	389	29	7.5%	52	13.4%	360	21	5.8%	21	5.8%
2010	360	18	5.0%	49	13.6%	387	20	5.2%	27	7.0%
2011	406	22	5.4%	62	15.3%	372	20	5.4%	19	5.1%
2012	434	28	6.5%	46	10.6%	356	24	6.7%	24	6.7%
2013	388	21	5.4%	50	12.9%	404	18	4.5%	34	8.4%
2014	424	22	5.2%	72	17.0%	383	22	5.7%	21	5.5%
2015	378	19	5.0%	51	13.5%	395	23	5.8%	22	5.6%
2016	403	34	8.4%	75	18.6%	374	22	5.9%	28	7.5%
2017	409	33	8.1%	66	16.1%	416	24	5.8%	21	5.0%

*P* for trend		0.682		0.165			0.815		0.780	

Body mass index (BMI) cut-offs were used to determine overweight and underweight children;

overweight boys and girls: BMI (kg/m^2^) >20.04 and >20.97 at age 10 years, and >23.26 and >23.89 at age 13, respectively.

underweight boys and girls: BMI (kg/m^2^) <14.57 and <14.39 at aged 10 years, and <16.52 and <16.17 at age 13, respectively.

The Cochran-Armitage test was used to evaluate secular trends.

**Figure 1.**
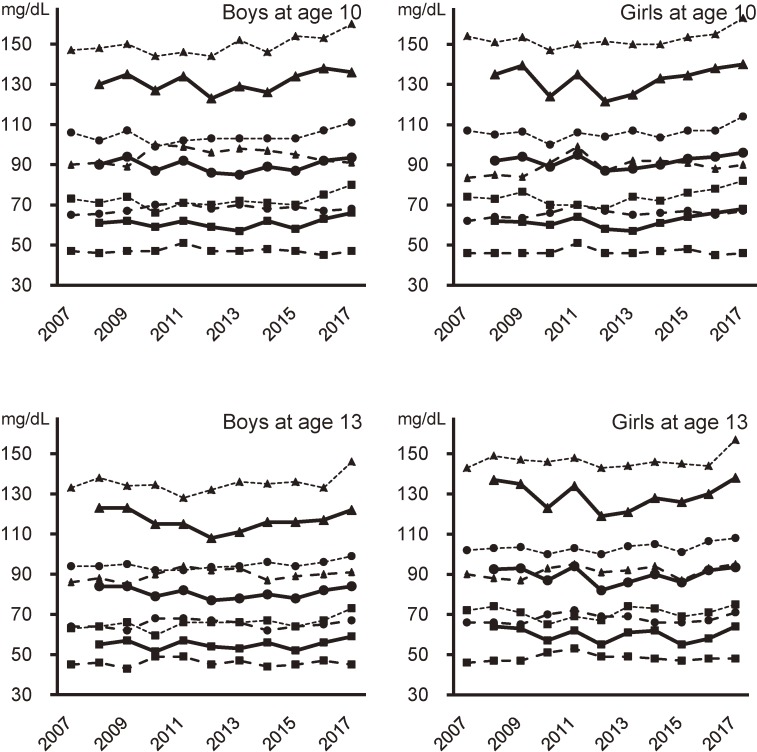
Secular trends in the percentiles of serum cholesterol levels. Triangles, 95th percentile; Circles, 50th percentile; Squares, 5th percentile; Solid lines, low-density lipoprotein cholesterol (LDL-C); Broken lines, high-density lipoprotein cholesterol (HDL-C); Dotted lines, non-high-density lipoprotein cholesterol (non-HDL-C). The Jonckheere-Terpstra test was used to evaluate secular trends, with calendar year as the explanatory variable and individual measurements as dependent variables. In children aged 10 years, significant increases were observed in LDL-C (*P* = 0.009 in boys, 0.014 in girls), HDL-C (*P* = 0.021 in boys, *P* < 0.001 in girls), and non-HDL-C (*P* < 0.001 in both sexes). In children aged 13 years, significant increases were observed in non-HDL-C in boys (*P* = 0.001) and girls (*P* < 0.001), and in HDL-C in girls (*P* = 0.017).

Figure 1 shows serum levels (95th, 50th, and 5th percentiles) of LDL-C, non-HDL-C, and HDL-C. LDL-C showed a significant positive association with calendar year in both boys and girls aged 10 years from 2008 through 2017. In addition, non-HDL-C showed a significant positive association with calendar year in both boys and girls aged 10 and 13 years from 2007 through 2017. Furthermore, HDL-C showed a significant positive association with calendar year in both boys and girls aged 10 years, and showed a significant increase in girls aged 13 years during the same period. T-C also showed a significant (*P* < 0.001) increase from 2007 through 2017, regardless of sex or age. The LDL-C to HDL-C ratio (LDL-C/HDL-C) showed no significant trends in both boys and girls aged 10 and 13 years from 2008 through 2017.

Table 3 shows the prevalence of adverse lipid levels from 2007 through 2017. No significant trends were observed during the study period in children with high LDL-C and those with low HDL-C, regardless of sex or age, while the prevalence of high non-HDL-C showed a significant increase in boys aged 10 and 13 years.

**Table 3.** Secular trends in children with dyslipidemia (numbers and proportions)

	Total	High LDL-C		High non-HDL-C		Low HDL-C		Total	High LDL-C		High non-HDL-C		Low HDL-C	
	Boys at age 10							Girls at age 10						
	
2007	387			23	5.9%	4	1.0%	400			37	9.3%	7	1.8%
2008	430	25	5.8%	26	6.0%	6	1.4%	391	28	7.2%	30	7.7%	2	0.5%
2009	452	33	7.3%	35	7.7%	2	0.4%	380	37	9.7%	34	8.9%	4	1.1%
2010	419	18	4.3%	20	4.8%	3	0.7%	432	12	2.8%	24	5.6%	7	1.6%
2011	461	34	7.4%	29	6.3%	3	0.7%	395	29	7.3%	32	8.1%	0	0.0%
2012	423	15	3.5%	21	5.0%	6	1.4%	420	13	3.1%	28	6.7%	4	1.0%
2013	434	18	4.1%	34	7.8%	5	1.2%	410	13	3.2%	29	7.1%	4	1.0%
2014	435	19	4.4%	25	5.7%	3	0.7%	434	25	5.8%	32	7.4%	4	0.9%
2015	442	30	6.8%	36	8.1%	6	1.4%	420	32	7.6%	37	8.8%	2	0.5%
2016	430	39	9.1%	40	9.3%	3	0.7%	395	32	8.1%	35	8.9%	5	1.3%
2017	502	40	8.0%	60	12.0%	4	0.8%	412	37	9.0%	54	13.1%	4	1.0%

*P* for trend		0.080		<0.001		0.882			0.204		0.060		0.524	

	Boys at age 13							Girls at age 13						
	
2007	395			8	2.0%	8	2.0%	373			16	4.3%	2	0.5%
2008	382	11	2.9%	13	3.4%	6	1.6%	346	24	6.9%	22	6.4%	3	0.9%
2009	389	10	2.6%	10	2.6%	8	2.1%	360	25	6.9%	21	5.8%	3	0.8%
2010	360	3	0.8%	8	2.2%	0	0.0%	387	13	3.4%	21	5.4%	1	0.3%
2011	406	7	1.7%	8	2.0%	3	0.7%	372	24	6.5%	22	5.9%	1	0.3%
2012	434	5	1.2%	10	2.3%	8	1.8%	356	9	2.5%	14	3.9%	1	0.3%
2013	388	7	1.8%	10	2.6%	2	0.5%	404	11	2.7%	20	5.0%	3	0.7%
2014	424	10	2.4%	12	2.8%	7	1.7%	383	16	4.2%	21	5.5%	2	0.5%
2015	378	8	2.1%	13	3.4%	5	1.3%	395	15	3.8%	20	5.1%	4	1.0%
2016	403	10	2.5%	13	3.2%	3	0.7%	374	19	5.1%	17	4.5%	2	0.5%
2017	409	10	2.4%	21	5.1%	7	1.7%	416	33	7.9%	37	8.9%	5	1.2%

*P* for trend		0.723		0.027		0.449			0.842		0.243		0.359	

LDL-C, low-density lipoprotein cholesterol; HDL-C, high-density lipoprotein cholesterol.

2011 Expert Panel cut-offs (high LDL-C, ≥130 mg/dL; high non-HDL-C, ≥145 mg/dL; low HDL-C, <40 mg/dL) were used.

The Cochran-Armitage test was used to evaluate secular trends.

## DISCUSSION

This is the first study to report secular trends of LDL-C in Japanese children. We found no remarkable changes in the prevalence of adverse LDL-C (≥130 mg/dL) or HDL-C (<40 mg/dL) in a population of school-aged children in Fukuroi City during the past decade, while the prevalence of high non-HDL-C increased. With regard to serum cholesterol levels, both HDL-C and LDL-C slightly increased in children aged 10 years for both sexes, and HDL-C slightly increased in girls aged 13 years.

The Circulatory Risk in Communities Study has reported that serum levels of LDL-C were positively associated with risk of coronary heart disease in a Japanese population.^24^ On the other hand, dyslipidemia, including high LDL-C, in adults often emerges during childhood,^8^ and the Expert Panel on Integrated Guidelines for Cardiovascular Health and Risk Reduction in Children and Adolescents strongly recommends cardiovascular health and risk reduction in children and adolescents.^14^ However, no previous study has examined secular trends in LDL-C among Japanese schoolchildren. In the present study, the Fukuroi population aged 10 years showed a yearly increase in serum LDL-C levels, with an accompanying increase in HDL-C levels. The present study also showed no significant trends in LDL-C/HDL-C in both boys and girls aged 10 and 13 years. The increase in HDL-C levels might have counteracted the effects of increased LDL-C levels in our study population. In addition, there were no remarkable trends in the prevalence of adverse lipid levels (ie, high LDL-C or low HDL-C). In Japan, nationwide surveys regarding serum T-C levels were conducted in 1960, 1970, 1980, and 1990, revealing a yearly increase in serum T-C levels among children and adolescents.^17^ More recently, our previous study found no significant trends in T-C, non-HDL-C, and HDL-C levels from 1993 to 2008 in a 10-year-old population in Iwata City (a city adjacent to Fukuroi City).^19^ The Japanese National Nutrition Survey reported that the total energy consumption per capita among 7- to 14-year-old children was 1,985 kcal/day in 2007 and 1,976 kcal/day in 2016, and that there were no remarkable differences in body height and weight among 13-year-old children between 2007 and 2016.^25^ Similar to these reports, the present study revealed no significant trends in BMI and the prevalence of overweight, while the prevalence of underweight showed a decrease in girls at age 10. To predict the prevalence of adult cardiovascular disease in the future, a continuous survey regarding blood lipids among children will be necessary.

With respect to serum LDL-C levels, both 10-year-old boys and girls showed significant increases during the study period; however, no significant trends were observed in 13-year-old children. These results suggest that there may be an age-related difference in trends of serum cholesterol. A possible explanation can be provided in terms of susceptibility, which is defined as a capacity characterized by biological factors that can modify the effect of a specific exposure.^26^ It is recognized that children are more susceptible to environmental exposure and have a greater potential for adverse health effects than adults.^27^^,^^28^ This may explain why environmental effects on serum lipids may be smaller during juvenile and adolescent periods compared to previous growth stages. Susceptibility may be one reason for the age-related difference in trends of serum cholesterol.

The present study has notable strengths. First, this study is the first to report trends in LDL-C in Japanese children. Childhood trends in serum lipids, which include not only T-C, HDL-C, and non-HDL but also LDL-C, provide additional information to predict subsequent cardiovascular disease trends in adults. Second, the source population of the present study comprised all children in Fukuroi City aged 10 and 13 years. Moreover, the analyzed population was 93.8% of the source population. Third, data were obtained from annual health examinations conducted during a consistent period (April to June) from 2007 through 2017. Reportedly, dynamic changes in serum lipids are observed during puberty, and serum lipid concentrations decrease with pubertal growth.^23^ If data of serum lipids were collected at the same age, this might have helped remove the effect of growth on serum lipid levels. However, since blood sampling was performed during the 3-month period in the present school screening, the effect of growth on the lipids levels could not be removed completely. In contrast, there are also some limitations worth noting. First, our data were obtained from only one city in Japan, so the data were not randomly selected from throughout Japan. Yet, according to a nationwide report of the School Health Examination Survey by the Ministry of Education, Culture, Sports, Science and Technology, Japan (conducted from April through June in 2016), at age 10, mean height was 139 cm in boys and 140 cm in girls, and mean body weight was 34 kg in both boys and girls.^29^ At age 13, mean height was 160 cm in boys and 155 cm in girls, and mean body weight was 49 kg in boys and 47 kg in girls.^29^ Thus, mean height and weight are similar between the Fukuroi population and the general population in Japan. If there are no remarkable differences in characteristics between the Fukuroi population and the overall Japanese population, trends of the Japanese population may be inferred from trends of the Fukuroi population. Second, many factors related to serum lipids, including physical activity and dietary habits, were not considered in the present study. Further investigation is warranted; in particular, a study involving several cities throughout Japan and a large-scale survey of lifestyle in children to examine future cardiovascular disease trends in Japanese adults would be useful.

In conclusion, we found no remarkable changes in the prevalence of adverse LDL-C levels from 2008 through 2017 and HDL-C levels from 2007 through 2017 in a population of school-aged children in Fukuroi City, while the prevalence of high non-HDL-C increased from 2007 through 2017. Among boys and girls aged 10 years, both serum levels of LDL-C and HDL-C slightly increased during the past decade. Serum HDL-C levels also slightly increased in girls aged 13 years during the same period.
